# Glutathione: Antioxidant Properties Dedicated to Nanotechnologies

**DOI:** 10.3390/antiox7050062

**Published:** 2018-04-27

**Authors:** Caroline Gaucher, Ariane Boudier, Justine Bonetti, Igor Clarot, Pierre Leroy, Marianne Parent

**Affiliations:** Université de Lorraine, CITHEFOR, F-54000 Nancy, France; ariane.boudier@univ-lorraine.fr (A.B.); justine.bonetti@univ-lorraine.fr (J.B.); igor.clarot@univ-lorraine.fr (I.C.); pierre.leroy@univ-lorraine.fr (P.L.); marianne.parent@univ-lorraine.fr (M.P.)

**Keywords:** glutathione, antioxidant, redox signaling, nanotechnologies, repletion, targeted drug delivery

## Abstract

Which scientist has never heard of glutathione (GSH)? This well-known low-molecular-weight tripeptide is perhaps the most famous natural antioxidant. However, the interest in GSH should not be restricted to its redox properties. This multidisciplinary review aims to bring out some lesser-known aspects of GSH, for example, as an emerging tool in nanotechnologies to achieve targeted drug delivery. After recalling the biochemistry of GSH, including its metabolism pathways and redox properties, its involvement in cellular redox homeostasis and signaling is described. Analytical methods for the dosage and localization of GSH or glutathiolated proteins are also covered. Finally, the various therapeutic strategies to replenish GSH stocks are discussed, in parallel with its use as an addressing molecule in drug delivery.

## 1. Introduction: Biochemical Implication of Glutathione in Redox Homeostasis

**Figure 1:**
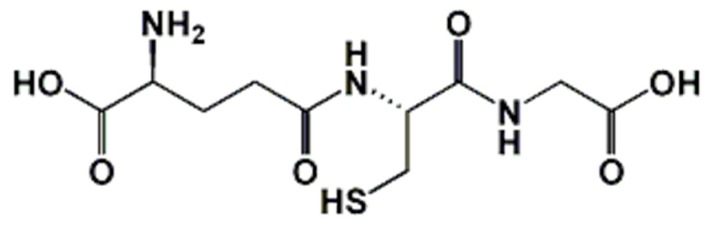
Condensed structural chemical formula of glutathione (IUPAC name: (2S)-2-amino-4-{[(1R)-1-[(carboxymethyl)carbamoyl]-2-sulfanylethyl] carbamoyl}butanoic acid).

Reduced glutathione (GSH; Figure 1) is the main low-molecular-weight thiol-containing peptide present in most living cells from bacteria to mammals (except some bacteria and amoebae) [1]. Since its discovery 130 years ago in baker’s yeast (*Saccharomyces cerevisiae*) by J. de Rey Pailhade, who named it “philothion”, many works have tried to establish and have elucidated its pivotal role in aerobic life. Its structure and redox role were established by Sir Frederick Gowland Hopkins in 1922 [2]. Glutathione was first claimed to be a dipeptide, and then a tripeptide, that is, γ-l-glutamyl-l-cysteinylglycine [3].

**Figure 2:**
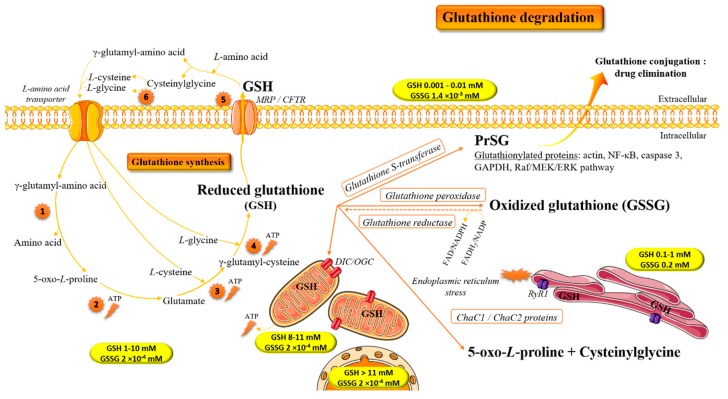
The homeostasis of reduced (GSH) and oxidized/disulfide (GSSG) glutathione within cells [12,13,14,15,16,17].

In the 1970s, the cellular glutathione cycle, involving two ATP-dependent enzymatically catalyzed steps for its synthesis, was established by Meister (Figure 2) [4,5]. The first enzyme, the γ-glutamylcysteinyl ligase (EC 6.3.2.3; GCL), is a heterodimeric rate-limiting enzyme. In animals and humans, the transcription of the GCL gene is regulated by nuclear factor erythroid-derived 2-like 2 (NFE2L2), which is sensitive to oxidative stress. Indeed, NFE2L2 activates the transcription of genes under the control of the antioxidant responsive element in various cell types [6]. The second enzyme, the glutathione synthase (EC 6.3.2.2; GS), catalyzes the formation of the covalent bond between the glycine residue and the γ-glutamyl-cysteine dipeptide. The rate of GSH synthesis is controlled by the cell content of l-cysteine [5] and ATP, and the ratio between the two subunits of the GCL [7], as well as its feedback inhibition by GSH [8] and oxidative stimulation of GCL enzymatic activity [9]. Although all cell types synthesize GSH, the main GSH source in the body remains the liver [10,11].

The synthesis of GSH is controlled by enzymes of Meister’s cycle: **1.** γ-Glutamyl cyclotransferase. **2.** 5-Oxoprolinase. **3.** γ-Glutamyl-cysteine ligase. **4.** GS; **5.** γ-Glutamyl transferase. **6.** Dipeptidase. These last two enzymes contribute to extracellular GSH catabolism, whereas glutathione-specific γ-glutamylcyclotransferases, the ChaC1 and ChaC2 proteins (EC 4.3.2.7) handle its intracellular catabolism. After synthesis, GSH is distributed between intracellular organelles by transporters such as the dicarboxylate carrier (DIC) and the oxoglutarate carrier (OGC) on the mitochondria and the ryanodine receptor type 1 (RyR1) on the endoplasmic reticulum. The multidrug resistance-associated proteins (MRPs) and the cystic fibrosis transmembrane conductance regulator (CFTR) are in charge of GSH cell efflux.

The γ-bond between the two amino acids, glutamic acid and cysteine, provides peculiar characteristics, such as insusceptibility to proteolysis. Moreover, the thiol-containing cysteine residue confers redox catalytic properties. Glutathione indeed resists hydrolysis of most of the proteases and peptidases, except γ-glutamyltransferase (EC 2.3.2.2; GGT) and the enzymes from the ChaC family. GGT, an exofacial plasmic membrane, is able to transfer the γ-glutamyl residue of glutathione to an acceptor (e.g., peptides) and release the dipeptide cysteinylglycine. It is the only known enzyme able to catabolize both GSH and GSH adducts (e.g., oxidized glutathione, glutathione *S*-conjugates and glutathione complexes) [18,19]. The glutathione catabolism is controlled by GGT, and the released cysteinylglycine is next degraded by a dipeptidase; then resulting free amino acids enter the cell to permit the de novo GSH synthesis. GGT is a glycosylated glycoprotein that shows multiple sites of *N*-glycosylation depending on the species and localization of the enzyme. For example, seven *N*-glycosylation sites have been identified in human GGT [20]. Furthermore, GGT is redox-regulated at the transcriptional and translational levels as well as in its enzymatic activity [20,21]. Other cytosolic enzymes from the ChaC family have recently been reported to catalyze the cleavage of glutathione. The ChaC enzymes, ChaC1 and ChaC2, are cytosolic glutathione-specific γ-glutamylcyclotransferases, cleaving the amide bond via transamidation using the α-amine of the L-glutamyl residue, releasing it as cyclic 5-oxo-l-proline and cysteinylglycine dipeptide. The ChaC1 and ChaC2 enzymes show around 50% of sequence homology and specifically degrade glutathione but no other γ-glutamyl peptides or oxidized glutathione [22,23]. The ChaC2 proteins are characterized by a lower catalytic efficiency than ChaC1 and are constitutively expressed [23]. ChaC1 is a proapoptotic enzyme under the regulation of CHOP (C/EBP homologue protein) transcription factor during the unfolding protein response of endoplasmic reticulum stress [24].

Therefore, the intracellular and extracellular GSH concentrations are determined by the balance between its synthesis and catabolism, as well as by its transport between the cytosol and the different organelles or the extracellular space. The mechanisms of GSH transport into the mitochondria and the endoplasmic reticulum have not been established. However, the DIC and the OGC seem to contribute to the transport of GSH across the mitochondrial inner membrane [25], and RyR1 participates in the accumulation of GSH in the endoplasmic reticulum [26,27]. Although some of the GSH synthesized within cells is delivered to intracellular compartments, much of it is exported across the plasma membrane into the extracellular spaces, especially under oxidative stress [28]. Plasma membrane proteins such as MRPs and the CFTR are implicated in the GSH export from cells essentially under oxidative stress [29,30]. The ability to export both GSH and oxidized derivatives of GSH endows these transporters with the capacity to directly regulate the cellular thiol-redox status and therefore the ability to influence many key signaling and biochemical pathways.

The physiological functions of glutathione range from (i) the maintenance of cysteine under a reduced state within proteins; (ii) the formation of a cysteine pool; (iii) the metabolism of oestrogens, leukotrienes, and prostaglandins and the production of deoxyribonucleotides; (v) the maturation of iron–sulfur clusters in proteins; and (vi) the transduction of redox signals to the cell transcription machinery; to (vii) the maintenance of the cell redox potential [31]. Indeed, as a result of its ability to exist in different redox states, GSH is implicated in processes of the maintenance and regulation of the thiol-redox status [32].

## 2. Glutathione and Antioxidant/Pro-Oxidant Properties

Even if the glutathione reducing power was described early on by reacting with the redox probe methylene blue [2], its redox role corresponding to a Nernstian response was clearly established in this century [33]. From a thermodynamic point of view (Gibbs free energy of redox reactions at the equilibrium, excluding enzyme activity in the redox buffering), two molecules of GSH simultaneously exchange two electrons and two protons with its disulfide form (GSSG). Taking into account the high concentration of glutathione in cells (in the millimolar range) and the high proportion of its reduced form (more than 98% in healthy cells), the GSSG/2 GSH redox couple is considered as the main cellular redox buffer. The standard potential value *E°* of the couple GSSG/2 GSH is equal to +197 mV, and because of its pH dependence according to the Nernst equation (Equation (1)), its apparent potential *E°′* at the physiological pH is equal to −240 mV.

(1) $$E{{}^{\circ}}^{\prime }=E{}^{\circ}-0.059\times pH\times \log\frac{{\left[GSH\right]}^{2}}{\left[GSSG\right]}$$

The redox metabolism of the cells depends also on redox enzymes working under steady-state conditions instead of the equilibrium defining thermodynamic systems. From this point of view, the enzymes’ reaction rate and kinetic constants (k_cat_ and K_m_ values), as well as GSH concentration and localization, are fundamental to highlight kinetic competition and to completely understand the GSH metabolism [17,34].

Therefore, the concentration of GSH as well as the [GSH]/[GSSG] ratio is a marker of oxidative stress and of the cell redox homeostasis. Regarding GSH reducing properties, it plays the role of an antioxidant as a scavenger of electrophilic and oxidant species either in a direct way or through enzymatic catalysis: (i) it directly quenches reactive hydroxyl free radicals, other oxygen-centered free radicals, and radical centers on DNA as well as on other biomolecules such as methylglyoxal and 4-hydroxynonenal; and (ii) GSH is the co-substrate of glutathione peroxidase (EC 1.11.1.9; GPx), permitting the reduction of peroxides (hydrogen and lipid peroxides) and producing GSSG. In turn, GSSG is reduced to 2 GSH by using NADPH reducing equivalents and glutathione disulfide reductase (EC 1.6.4.2; GR) catalysis. Electrophilic endogenous compounds and xenobiotics (drugs, pollutants and their phase I metabolites) are conjugated with GSH through activation by glutathione-*S*-transferases (EC 2.5.1.18; GSTs). The resulting conjugates are substrates of GGT, which initiates the mercapturic acid pathway and facilitates toxic elimination.

**Figure 3:**
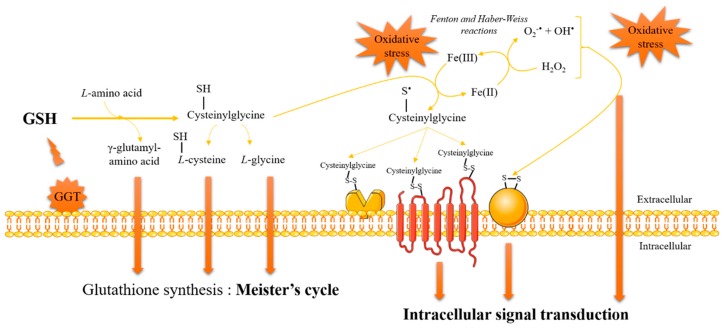
The pro-oxidant activity of γ-glutamyltransferase (GGT) adapted from [35].

The overall cellular redox homeostasis is aimed at maintaining harmful reactive oxygen and nitrogen species and GSSG at very low levels and GSH at a high level. However, GSH can play a pro-oxidant role, which occurs to a lesser extent than its antioxidant role. This process has been reported during the GSH catabolism via GGT catalysis (Figure 3) [35,36]. The mechanism relies upon a shift of pKa of the SH group in the cysteine residue between GSH (pKa of 8.7) and its breakdown product cysteinylglycine (pKa of 6.4). Consequently, the proportion of the more reactive thiolate form is higher in cysteinylglycine than in GSH at the physiological pH value: the reduction rate of ferric ions to ferrous ions is higher, and the production of hydroxyl radicals and superoxide anions through Fenton and Haber–Weiss reactions is more abundant. The GSH pro-oxidant effect occurs, at least initially, on the outer side of the plasma membrane inducing lipid peroxidation, which destabilizes the cell membrane structure. Then, pro-oxidant effects, which are not stopped by the antioxidant defense systems [36], are propagated inside the cell through a signaling process. The pro-oxidant action of GGT is linked to the presence of redox active metals in the extracellular space. The reactivity of free redox active metals is strongly prevented in vivo by complexation with ferritin, transferrin or ceruloplasmin, for example. In this regard, the GGT activity can be postulated as able to reduce and to promote the release of iron ions from transferrin [37,38] as well as copper ions from ceruloplasmin [39]. An excess production of reactive oxygen species through GSH catabolism will produce DNA damages [40,41] or trigger the lipid peroxidation, already documented in vitro for linoleic acid [42] and for low-density lipoproteins (LDLs). Indeed, LDLs’ oxidation process catalyzed during the reduction of iron (Fe^3+^ to Fe^2+^) is known to play a central role in atherogenesis and vascular damage. Moreover, thiol-containing residues such as cysteine and homocysteine are known to reduce Fe^3+^ and promote Fe^2+^-dependent LDL oxidation [43]. The γ-glutamate residue of GSH decreases the interactions between the thiol function of the cysteine residue and iron, precluding Fe^3+^ reduction and hence LDL oxidation [44,45]. The catabolism of GSH by GGT removing the γ-glutamate residue from the cysteine residue increases the iron reduction and LDL oxidation remarkably [38].

## 3. Glutathione and Redox Signaling

Glutathione plays a crucial role in cell signaling via two pathways: (i) the modification of the redox potential toward oxidative values linked to the GSH concentration decrease and/or GSSG increase can activate transcription factors, which provokes gene activation and the synthesis of proteins with antioxidant properties; and (ii) the formation of the disulfide bond between protein thiol groups (PSHs) and GSH generates mixed (protein/non-protein) disulfides, that is, *S*-glutathiolated proteins (PSSGs).

Three main redox systems, the GSSG/2 GSH couple, the NADP+/NADPH couple and the thioredoxin system, regulate the intracellular redox potential [33]. However, as the intracellular concentration of GSH is very high, the ratio of the concentration of GSSG and GSH is fundamental for signal transductions, such as in the cell-cycle regulation [33]. Depending on the conditions, the in vivo redox potential of the GSSG/2 GSH couple ranges from −260 to –150 mV [46]. In fact, shifting the GSSG/2 GSH ratio toward the oxidizing state (redox potential of up to −150 mV) reduces cell proliferation and increases apoptosis through the activation of several signaling pathways, including calcineurin, NF-κB, protein kinase B, c-Jun N-terminal kinase, apoptosis signal-regulated kinase 1, and mitogen-activated protein kinase [47]. Furthermore, the cellular redox environment fluctuates during the cell cycle. Indeed, the cellular GSH content is significantly higher in the G2 and M phases compared with G1, while cells in the *S*-phase show an intermediate redox state [48]. Pharmacologic and genetic manipulations of the cellular redox environment perturb normal cell-cycle progression [49,50].

The process *S*-glutathiolation, a reversible post-translational modification of proteins, may have a role in the protection of PSHs from irreversible oxidation and in the redox regulation of the protein function, serving for cell signaling [51]. Indeed, if the modified cysteine is critical for the protein function, the *S*-glutathiolation will also either inactivate the protein or compromise cellular functions. Several *S*-glutathiolation mechanisms have been proposed: the direct reaction of GSH with partially oxidized reactive PSHs (thiyl radicals or sulfenic acids), thiol–disulfide exchange between PSHs and GSSG or between PSHs and oxidized GSH (sulfenic acid (GSOH)), nucleophilic attack of a protein thiolate on *S*-nitrosoglutathione (GSNO), and finally, PSHs’ *S*-nitrosation followed by *S*-glutathiolation by GSH to yield the rate of mixed disulfides’ formation. Protein *S*-glutathiolation can also change the protein activity and have a role in redox signaling. Under normal physiological conditions, the glutathiolation status of some proteins is important for many vital functions such as actin polymerization, transcription factor activation, and apoptosis [51]. Glutaredoxin, whose major isoforms in mammals are Grx1, Grx2, and Grx5, as well as thioredoxin, catalyzes *S*-glutathiolation and deglutathiolation of proteins to protect SH-groups from oxidation and restore functionally active thiols [52].

## 4. Methodologies for Dosage of Glutathione/Glutathiolated Proteins

As glutathione plays a fundamental role in cellular homeostasis, the changes in the GSH/GSSG ratio and concentrations are especially important in the evaluation and diagnosis of many redox-related pathologies, such as cancers [53], neurodegenerative diseases [54] or stroke [55], and cardiovascular diseases [56]. Glutathione in biological fluids (e.g., plasma) is known to demonstrate great instability, with a half-life of about 20 min [57]. The methodology used for GSH or GSSG quantification is therefore essential to achieve (i) the specificity required to discriminate between the various forms of glutathione and other endogenous thiols present in the biological matrices (cells, tissues, fluids, etc.), as well as (ii) the mandatory selectivity to separate the reduced and oxidized glutathione forms. Another important methodological criterion is sensitivity. For GSH, this parameter is often not critical because of its high concentration in cells (from 1 to 10 mM) and plasma (from 1 to 6 µM [58]). This will often be a problem for GSSG, which is present at low concentrations under physiological conditions, at around 1% of intracellular GSH levels.

At present, there are many methods for evaluating GSH and GSSG in biological samples, from the classical enzymo-colorimetric method developed by Tietze in 1969 [59], to spectrophotometric [60] or spectrofluorimetric [61] methods. The lack of chromophores and fluorophores in the glutathione family has led to the development of numerous derivatization methods (e.g., *N*-pyrenemaleimide or *O*-phthalaldehyde (OPA) [62,63,64,65,66]) or the use of electrochemical methods [67], mass spectrometry [68], chemiluminescence [69], nuclear magnetic resonance [70] or surface-enhanced Raman scattering [71]. Separation methods are also in full extension for this type of application, using chromatographic techniques (ultra-performance liquid chromatography [72], high-performance liquid chromatography [73], and gas chromatography [74]) or electrophoretic techniques [75], such as, for example, the quality control of GSH produced by microorganisms for pharmaceutical use [76].

The different methodologies mentioned above can be very effective but require specific equipment and an important analysis time, as well as peculiar sample processing or treatment. In this context, the use of nanotechnologies (essentially gold and silver metallic nanoparticles (NPs)) appears to be an interesting solution to quantify glutathiolated species. Gold and other metallic species are indeed characterized by an important avidity to bind sulfhydryl compounds [77,78,79]. In an organism, these atoms, including those under a NP state, form a bond with the thiol function of glutathione, even if the sulfur–metal interfacial chemistry remains controversial in the literature [77,80]. Over the past years, quantification on the basis of NPs has been largely developed to evaluate GSH in intracellular and/or extracellular concentrations in various biological matrices such as plasma, urine or saliva. Many colorimetric methods using gold NPs (AuNPs) (sensor or probes) [81] have been developed on the basis of their high molar absorbance coefficient associated with a specific plasmonic resonance-band shift (the maximum wavelength moves from a dispersed to an aggregated state or vice versa; see, e.g., [82]). The development of such nanosystems is very interesting but can lead to many problems of interference as a result, in particular, of chemical structures close to glutathione in large quantities in the body, such as cysteine or homocysteine [83]. Current methods make it possible to reach nanomolar concentrations, such as capillary electrophoresis coupled laser-induced fluorescence, as described by Shen et al. [84]. To a lesser extent, silver NPs are also used to evaluate glutathione in a biological medium with the same advantages as AuNPs described previously [85,86]. Other, more marginal examples of NPs developed specifically to quantify GSH or GSSG, such as NPs of alumina or zinc, can be found in the literature [87,88].

All the examples mentioned above show that nanotechnology is the future of the determination of many biological molecules, glutathione clearly being a part of it.

## 5. Repletion of Glutathione: Therapeutic Opportunities and Challenges

**Figure 4:**
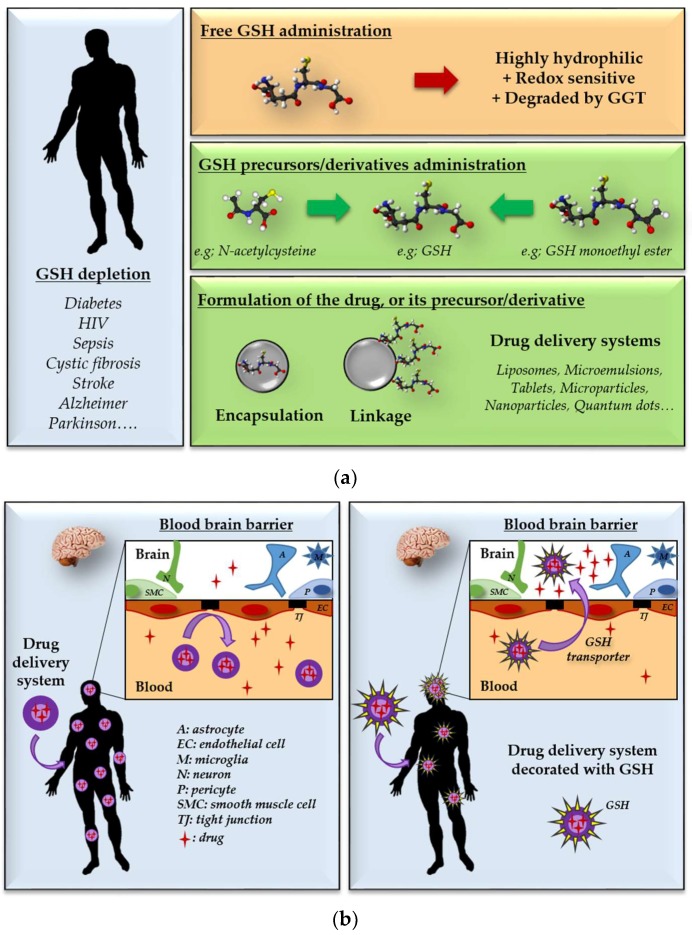
Schematic representations of the links between nanotechnologies and glutathione. (**a**) How nanotechnologies can help to reconstitute the glutathione pool? (**b**) How GSH can help nanotechnologies to reach their target? The example of Brain delivery.

In cancer therapy, the depletion of glutathione has emerged as a valuable strategy to increase the sensitivity of cancer cells to radiations and toxic drugs, especially for aggressive and/or metastatic cancers [89,90,91]. However, a great majority of diseases (diabetes [92], cardiovascular diseases [56], HIV/AIDS [93], sepsis [94], cystic fibrosis [95], stroke [55], and brain disorders such as Alzheimer’s and Parkinson’s diseases or schizophrenia [96,97,98]), are associated with a decrease in GSH, combined with various oxidative stress states. Similarly, it has been demonstrated that the GSH antioxidant defenses of the body decrease linearly with age (oxidation of the GSSG/2 GSH couple in plasma: +0.7 mV/year after 45 years of age [99]), leaving us vulnerable to many age-related diseases. As a result, therapeutic strategies to restore the GSH pool are needed, ideally through oral administration for such chronic conditions. This is especially challenging because of the physicochemical properties of GSH (low partition coefficient; PubChem’s calculated log P = −4.5) and its high degradation rate in the gastrointestinal tract through bacterial and epithelial GGT catalysis [100]. Two complementary approaches might increase GSH bioavailability: one is based on chemistry and the other is based on drug delivery technology (Figure 4a).

The administration of prodrugs of GSH (or GSH precursors such as cysteine) is a first option. However, cysteine cannot be administered directly because of its toxicity and instability [101]; therefore precursors of cysteine, for example, the well-known *N*-acetylcysteine (NAC), are used. NAC is a potent antioxidant and an established antidote for acetaminophen overdose (which depletes hepatic GSH). Both parenteral and oral administrations are approved by the Food and Drug Administration for this indication, with different therapeutic schemes: loading dose followed by 17 additional doses over 72 h for oral NAC versus loading dose and 2 additional doses over 21 h for parenteral NAC [102]. Although several human clinical trials have investigated the potential of oral NAC to replenish GSH in chronic depletions (e.g., in HIV patients), all have failed to give sufficient benefits to gain regulatory approval, even at high doses (up to 2 g/day) [103]. These results can be explained by different mechanisms of GSH depletion between acute and chronic conditions and/or by the low NAC bioavailability when given per os (6–10% [104]). To improve tissue distribution, lipophilic derivatives of NAC were prepared: they gave encouraging results in cells (NAC amide [105]) or after peritoneal (NAC amide [106]) or oral administration in rats (NAC ethyl ester [107]). This strategy was also extended to GSH, creating more lipophilic derivatives through esterification. Glutathione esters (mainly mono- and di-methylesters) have been investigated as potential oral delivery compounds because they present a higher hydrophobicity and less sensitivity toward GGT degradation [108,109,110,111], but no conclusive data on their ability to restore the GSH pool in humans are currently reported in the literature. Furthermore, other cysteine or glutathione precursors have been evaluated with varying results: l-methionine; *S*-adenosylmethionine (SAMe) [112]; l-2-oxothiazolidine-4-carboxylate (OTC, procysteine), which is enzymatically converted to cysteine within liver cells [113]; 2-(RS)-n-propylthiazolidine-4(R)-carboxylic-acid (PTCA); d-ribose-l-cysteine; l-cysteine-glutathione mixed disulphide [114]; and γ-glutamylcysteine [115].

The second option for improving the bioavailability of GSH (or its derivatives) is to use a drug delivery system. In the literature, GSH has been encapsulated into various galenic forms for oral administration: liposomes [116], water-in-oil microemulsions [117], pellets of montmorillonite and glutathione [118], polymeric NPs and microparticles prepared with natural or synthetic polymers [119,120,121], hydrogels [122], and mucoadhesive films for sublingual delivery [123], as well as orobuccal tablets in combination with l-cystine, vitamin C and selenium [124]. Only this latter form has been tested per os in humans, with promising results: the GSH blood level increased significantly with time after administration of this tablet containing 250 mg of GSH to 15 healthy volunteers (both sexes, 20–40 years old) [124]. Aside from improving bioavailability, an additional benefit of formulations is to increase patient compliance while hiding the undesirable organoleptic (odor and flavor) properties of the thiol drugs.

The GSH can also be chemically linked to the carrier surface (instead of being passively encapsulated into the carrier). The main problem encountered with GSH conjugation on molecules remains the preservation of the thiol function, that is, to avoid formation of a disulfide. Many works use GSH as a stabilizer for metallic (mainly gold or silver) NPs [125,126,127] or nanoclusters [128] by the interaction between sulfur and metallic atoms, as previously explained. However, this prevents their use for the peptide repletion event if these particles seem to be well-tolerated by cells and the organism [129]. Only few works have reported the synthesis of GSH conjugates with a preservation of its antioxidant property. Two main strategies were led, grafting GSH either to polymers used as raw material for NP preparation, or to preformed NPs. First, GSH was covalently linked to chitosan [130] and polyethylene glycol [131]. Yields of grafting were 99% for polyethylene glycol–GSH oligomers [131] and 111 µmol GSH/g of functionalized chitosan [130]. In an in vitro model of oxidative stress (human brain neuroblastoma cells’ SH-SY5Y cell line; exposed to H_2_O_2_ for 24 h), cells pre-treated by oligomers (polyethylene glycol conjugated to GSH) were protected from oxidative damage [131]. Second, GSH was anchored to core shell (CdSe/ZnS) quantum dots (up to 40 GSH moieties per quantum dot [132]. These quantum dots were tested in an in vivo model using *Hydra vulgaris* [132]. The authors showed the localization of GSH binding proteins inside the animals after internalization, as a result of the fluorescent properties of the quantum dots. GSH was also conjugated to gold NPs (AuNPs) via a linker (lipoic acid), which was previously shown to passivate the NP surface [133,134], and which limited the access of the thiol function to the gold core [135]. In this work, the authors report the possible oligomerization of GSH during the process, which could explain the high grafting density (ca. 7500 GSH moieties per AuNP) on AuNPs. The preservation of GSH properties was demonstrated using classical redox tests. Compared to non-conjugated GSH AuNPs, an activity enhanced by factors of 10,000 and 36,000 was reported for AuNPs functionalized by GSH using 2,2′-azino-bis(3-éthylbenzothiazoline-6-sulphonic) acid (ABTS) and ferric reducing antioxidant power (FRAP), respectively [135].

To conclude, very few of these works have moved from research into clinic applications (apart from precursors/prodrugs of GSH such as NAC, and one GSH-containing orobuccal tablet), especially for oral GSH supplementation. The collaboration between the chemical and galenic approaches seems nevertheless to offer promising opportunities in the future.

## 6. GSH Decoration as a Tool for Targeted Drug Delivery Systems

Glutathione is now also being investigated as a molecular tool in the hand of chemists and pharmacists to specifically deliver drugs to the brain (Figure 4b) or to obtain controlled drug release in the intracellular compartment. These two aspects are discussed in this section.

### 6.1. Brain-Targeted Drug Delivery

**Figure 5:**
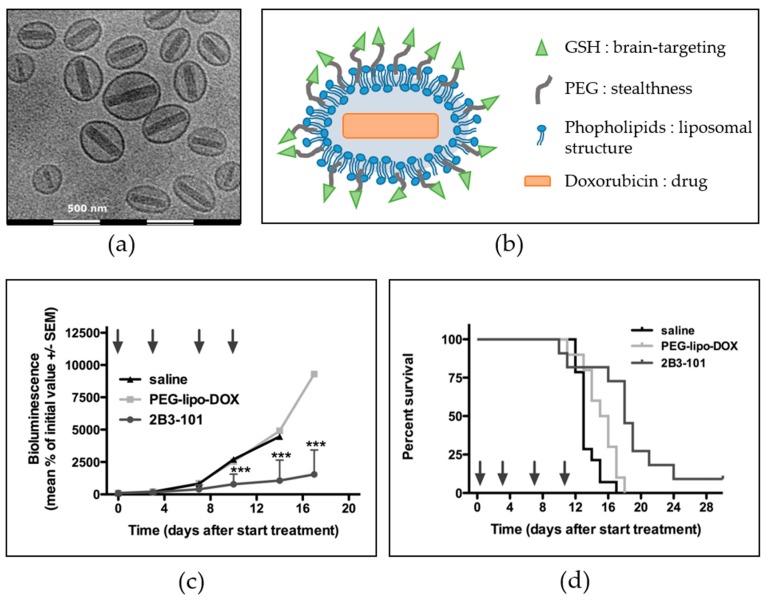
Brain targeting with reduced glutathione (GSH) decoration: example of the 2B3-101 product (GSH-coated–polyethylene glycol (PEG)ylated liposome containing doxorubicin). (**a**) Cryo-electron microscopy image of 2B3-101; (**b**) schematic representation of the liposomal structure; (**c**) inhibition of brain tumor growth in mice with experimental brain tumors; (**d**) increased survival of mice with experimental brain tumors. Animals received twice-weekly IV administrations (arrows on the graph) of saline (*n* = 14), 2B3-101 (*n* = 10) or PEGylated liposomal doxorubicin (PEG-lipo-DOX; *n* = 10), all at 5 mg/kg doxorubicin equivalents. *** *p* < 0.001, 2B3-101 vs saline and PEG-lipo-DOX; (**a**,**c**,**d**) are reproduced with permission from [144].

Drug delivery to the central nervous system represents one of the major pharmaceutical challenges, as the passage of macromolecules as well as 98% of small molecules is prevented by the blood–brain barrier under physiological conditions [136]. However, brain drug delivery can be achieved by taking advantage of the numerous endogenous specialized transport systems of this biological barrier. In the last years, GSH has emerged as a potential candidate to facilitate the receptor-mediated transcytosis of nanocarriers. The sodium-dependent (active) glutathione transporter is indeed present in all mammalian species, with a preferential expression in the central nervous system and the blood–brain barrier [137,138,139]. The conjugation of GSH on several pharmaceutical forms safely enhanced the delivery of various encapsulated drugs and nucleic acids to the brain. The story of G-technology, a liposomal system with a polyethylene glycol (PEG) coating modified with GSH, is developed as an example of the successful transfer from the pre-clinic to the clinic. Rip et al. evaluated the uptake of GSH-coated–PEGylated liposomes encapsulating carboxyfluorescein (an autoquenched fluorescent tracer) by brain endothelial cells, as well as their pharmacokinetic behavior and brain distribution after intraperitoneal or intravenous administration to rats [140]. The results demonstrated a temperature-dependent uptake of liposomes by the endothelial cells (about 2 times higher for GSH–PEG liposomes compared to uncoated liposomes). In rats, both administration routes gave comparable circulating levels and tissue distribution, and the brain levels of the fluorescent tracer were increased 4-fold by the GSH coating. This technology was used to deliver amyloid-targeting antibody fragments to the brain in a mouse model of Alzheimer’s disease after intravenous bolus [141]. The liposomes were prepared by the ethanol injection method, with cholesterol, mPEG-2000-1,2-distearoyl-sn-glycero-3-phosphoethanolamine, and different lipids: the brain accumulation was higher for egg-yolk phosphatidylcholine liposomes than 1,2-dimyristoyl-sn-glycero-3-phosphocholine liposomes. Maussang et al. studied the mechanisms of increased in vivo brain delivery of the model drug ribavirin when encapsulated into PEGylated liposomes conjugated with GSH, intravenously administered to rats [142]. They demonstrated that this brain-specific uptake was positively correlated with increasing amounts of GSH coating and involved a receptor-mediated mechanism. These GSH–PEG liposomes (G-technology) have also demonstrated brain targeting as well as therapeutic efficacy in murine models of brain cancer (2B3-101; drug: doxorubicin [143,144]) and neuroinflammation (2B3-201; drug: methylprednisolone [145]). As an example, the major results obtained with 2B3-101 in cells and animals are presented in Figure 5. The 2B3-201 product has recently completed a phase I trial in healthy volunteers [146], while the 2B3-101 product has completed a phase I/IIa trial in patients with various forms of brain cancer (ClinicalTrials.gov Identifier: NCT01386580 [147,148]) and is currently being tested in a phase II trial in patients with breast cancer and leptomeningeal metastases (ClinicalTrials.gov Identifier: NCT01818713).

GSH-coating is also under investigation to obtain the brain delivery of drugs encapsulated into NPs. Veszelka et al. have, for example, demonstrated on cells that GSH coating leads to a higher cell uptake of polystyrene NPs than biotin coating [149]. GSH-coated poly(lactic-*co*-glycolic acid) (PLGA)–PEG NPs containing docetaxel [150], doxorubicin [151] or paclitaxel [152] have also been developed and characterized for drug release, cytotoxicity and blood–brain barrier permeation. In animals, bovine serum albumin (BSA) NPs with a GSH ligand led to 10-fold higher brain concentrations of a neuroprotective agent than the drug solution, 5 h after intravenous injection [153]. In another study, a hydrophilic model drug was 3 times more concentrated in the brain after intravenous injection of GSH-coated BSA NPs, compared to the same uncoated NPs [154]. Finally, in a middle cerebral artery occlusion model of stroke, GSH-coated PLGA–PEG NPs containing the thyroid T3 hormone showed better therapeutic efficacy than T3 solution or uncoated NPs, on both tissue infarction (reduction of 58%, 34%, and 51%, respectively) and brain edema (reduction of 75%, 59% and 68%, respectively) [155]. Although these GSH-coated NPs are not already in use in clinical trials, they highlight that GSH-mediated brain targeting can be used with different galenic forms, for various drugs. Recently, this strategy has also been proved to be successful in vitro for gene delivery to brain endothelial cells [156], thus opening another wide field of applications.

### 6.2. Intracellular-Targeted Drug Delivery

Because of the difference in GSH concentrations between plasma and cytosol (0.001 mM vs 1 to 10 mM), the intracellular compartment is a more reductive environment than plasma. As a result, the difference in redox potential has been exploited by researchers to create stimuli-sensitive NPs. Using this strategy, a drug is not only protected in the blood flow but is also released specifically in this reductive environment, thus achieving intracellular targeting. Other external stimuli have been described on the basis of physical (light and electromagnetic field), chemical (pH and temperature) or even biochemical (enzyme) signals. To go further, there is growing research to develop sequential or simultaneous multi-stimuli responsive delivery systems. Interesting reviews on this topic can be found in the literature (e.g., [157,158,159]). As far as the redox stimulus is concerned, two main strategies are usually described, based either on the reduction of disulfide bounds structuring the NPs or on ligand exchange at the surface of inorganic NPs. An extensive literature deals with the design of new bio-reducible polymers (such as poly(ethylenimine), poly(amido amine) or polymers based on peptide or nucleic acid derivatives) [159,160]. In these works, the authors use the reduction of disulfide bounds (introduced into the polymer structure) by intracellular GSH to trigger the drug release through NP disorganization or disassembly. Another approach focuses on di-selenide or carbon–selenide bonds, which are more sensitive to the GSH concentration than disulfide bonds [161]. Chemical and pre-clinical proofs of concept have been brought forward for applications in gene silencing and imaging [162], as well as in the delivery of genes [160], proteins and anti-proliferative drugs [159,162]. None of these objects has nevertheless reached the clinical level so far.

**Figure 6:**
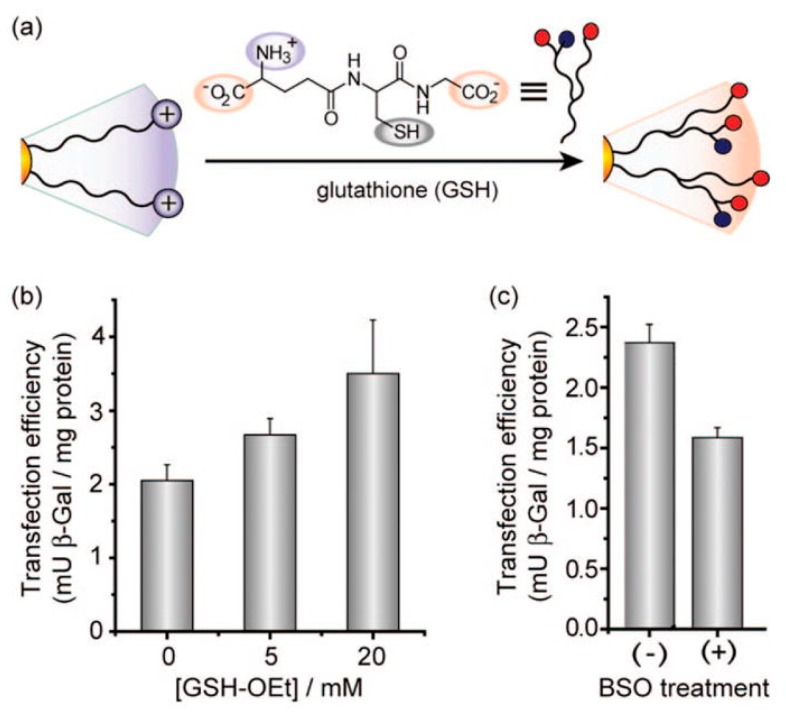
Example of ligand exchange strategy involving reduced glutathione (GSH) for targeted intracellular gene delivery. (**a**) Scheme of the ligand exchange reaction between native cationic ligands and cellular glutathione on gold nanoparticles’ surface. (**b**) Elevation in transfection level depending on dose of glutathione monoester (GSH–Oet). Monkey kidney cells were preincubated with GSH–Oet for 1 h then washed prior to transfection, to transiently increase the GSH level. (**c**) Decrease in transfection efficiency upon l-buthionine-[*S*,*R*]-sulfoximine (BSO) treatment. Cells were plated in BSO-containing (2 mM) media and incubated for 24 h. BSO is an inhibitor of γ-glutamylcysteine synthetase and thus suppresses baseline GSH production. Reprinted with permission from [163]. Copyright (2008) American Chemical Society.

Apart from disulfide reduction by GSH, another strategy using ligand exchange at the surface of AuNPs has been described (Figure 6). As previously explained, there is a strong avidity of AuNPs towards the thiol function of GSH. After internalization of AuNPs into the cellular cytoplasm, the drug grafted on the surface of the gold core is progressively released and replaced by GSH through thiol–gold binding. This was used to deliver the drug (e.g., paclitaxel) or for cell imaging [162,163]. However, intracellular GSH may be depleted, which could lead to oxidative stress in addition to other redox side-effects that may be induced by AuNPs [164].

## 7. Conclusions

Since its discovery, GSH has been shown to play ubiquitous roles in most living cells, from prokaryotic to eukaryotic organisms. GSH was defined as the intracellular redox buffer, and its major function, either free or associated to proteins, is tightly connected to redox reactions, mainly acting as a reductant versus oxygen and its derived reactive species. From physico-chemical and biochemical points of view, GSH redox properties are well defined and act in cell signaling through post-translational modifications. Disturbance of redox homeostasis related to the depletion of GSH has been shown more and more to be implicated in many pathophysiological states, opening a means for its use as a drug. Glutathione has clearly penetrated fields other than biology, such as therapeutics, with associated nanotechnology approaches for improving its bioavailability and targeting ability. Indeed, growing research considers GSH not only as a drug, but also as a tool for stimuli responsive in drug delivery systems.

## Abbreviations

ABTS	2,2′-Azino-bis(3-éthylbenzothiazoline-6-sulphonic) acid
AuNP	Gold nanoparticle
BSA	Bovine serum albumin
BSO	l-Buthionine-[*S*,*R*]-sulfoximine
CFTR	Cystic fibrosis transmembrane conductance regulator
DIC	Dicarboxylate carrier
FRAP	Ferric reducing antioxidant power
GCL	γ-Glutamylcysteinyl ligase
GGT	γ-Glutamyltransferase
GPx	Glutathione peroxidase
GR	Glutathione disulfide reductase
GS	Glutathione synthase
GSH	Reduced glutathione
GSH-Oet	Glutathione monoester
GSNO	*S*-Nitrosoglutathione
GSOH	Glutathione sulfenic acid
GSSG	Disulfide glutathione
GSTs	Glutathione-*S*-transferases
LDL	Low-density lipoprotein
MRP	Multidrug resistance-associated protein
NAC	*N*-Acetylcysteine
NFE2L2	Nuclear factor erythroid-derived 2-like 2
NP	Nanoparticle
OGC	Oxoglutarate carrier
OPA	*O*-Phthalaldehyde
OTC	l-2-Oxothiazolidine-4-carboxylate
PEG	Poly(ethylene)glycol
PLGA	Poly(lactic-co-glycolic acid)
PSHs	Protein thiol groups
PSSGs	*S*-Glutathiolated proteins
PTCA	2-(RS)-n-Propylthiazolidine-4(R)-carboxylic-acid
RyR1	Ryanodine receptor type 1
SAMe	*S*-Adenosylmethionine

## References

[1] Hamilton C.J., Arbach M., Groom M., Jacob C., Kirsch G., Slusarenko A., Winyard P., Burkholz T. (2014). Beyond Glutathione: Different Low Molecular Weight Thiols as Mediators of Redox Regulation and Other Metabolic Functions in Lower Organisms. Recent Advances in Redox Active Plant and Microbial Products.

[2] Gowland Hopkins F., Dixon M. (1922). On glutathione. II. A thermostable oxidation-reduction system. J. Biol. Chem..

[3] Hunter G., Eagles B.A. (1927). Glutathione: A critical study. J. Biol. Chem..

[4] Meister A., Tate S.S. (1976). Glutathione and related gamma-glutamyl compounds: Biosynthesis and utilization. Annu. Rev. Biochem..

[5] Meister A., Anderson M.E. (1983). Glutathione. Annu. Rev. Biochem..

[6] Baldelli S., Aquilano K., Ciriolo M.R. (2013). Punctum on two different transcription factors regulated by PGC-1α: Nuclear factor erythroid-derived 2-like 2 and nuclear respiratory factor 2. Biochim. Biophys. Acta.

[7] Chen Y., Shertzer H.G., Schneider S.N., Nebert D.W., Dalton T.P. (2005). Glutamate cysteine ligase catalysis: Dependence on ATP and modifier subunit for regulation of tissue glutathione levels. J. Biol. Chem..

[8] Taylor C.G., Nagy L.E., Bray T.M. (1996). Nutritional and hormonal regulation of glutathione homeostasis. Curr. Top. Cell. Regul..

[9] Krejsa C.M., Franklin C.C., White C.C., Ledbetter J.A., Schieven G.L., Kavanagh T.J. (2010). Rapid Activation of Glutamate Cysteine Ligase following Oxidative Stress. J. Biol. Chem..

[10] Lauterburg B.H., Adams J.D., Mitchell J.R. (1984). Hepatic glutathione homeostasis in the rat: Efflux accounts for glutathione turnover. Hepatology.

[11] DeLeve L.D., Kaplowitz N. (1991). Glutathione metabolism and its role in hepatotoxicity. Pharmacol. Ther..

[12] Cooper A.J., Pinto J.T., Callery P.S. (2011). Reversible and irreversible protein glutathionylation: Biological and Clinical aspects. Expert Opin. Drug Metab. Toxicol..

[13] Pineda-Molina E., Klatt P., Vázquez J., Marina A., García de Lacoba M., Pérez-Sala D., Lamas S. (2001). Glutathionylation of the p50 subunit of NF-kappaB: A mechanism for redox-induced inhibition of DNA binding. Biochemistry.

[14] Huang Z., Pinto J.T., Deng H., Richie J.P. (2008). Inhibition of caspase-3 activity and activation by protein glutathionylation. Biochem. Pharmacol..

[15] Pastore A., Piemonte F. (2013). Protein glutathionylation in cardiovascular diseases. Int. J. Mol. Sci..

[16] Circu M.L., Aw T.Y. (2012). Glutathione and modulation of cell apoptosis. Biochim. Biophys. Acta.

[17] Deponte M. (2017). The Incomplete Glutathione Puzzle: Just Guessing at Numbers and Figures?. Antioxid. Redox Signal..

[18] Taniguchi N., Ikeda Y. (1998). Gamma-glutamyl transpeptidase: Catalytic mechanism and gene expression. Adv. Enzymol. Relat. Areas Mol. Biol..

[19] Ohkama-Ohtsu N., Radwan S., Peterson A., Zhao P., Badr A.F., Xiang C., Oliver D.J. (2007). Characterization of the extracellular gamma-glutamyl transpeptidases, GGT1 and GGT2, in Arabidposis. Plant J..

[20] Hanigan M.H. (2014). Gamma-glutamyl transpeptidase: Redox regulation and drug resistance. Adv. Cancer Res..

[21] Zhang H., Forman H.J. (2009). Redox regulation of gamma-glutamyl transpeptidase. Am. J. Respir. Cell Mol. Biol..

[22] Kumar A., Tikoo S., Maity S., Sengupta S., Sengupta S., Kaur A., Bachhawat A.K. (2012). Mammalian proapoptotic factor ChaC1 and its homologues function as γ-glutamyl cyclotransferases acting specifically on glutathione. EMBO Rep..

[23] Kaur A., Gautam R., Srivastava R., Chandel A., Kumar A., Karthikeyan S., Bachhawat A.K. (2017). ChaC2, an Enzyme for Slow Turnover of Cytosolic Glutathione. J. Biol. Chem..

[24] Mungrue I.N., Pagnon J., Kohannim O., Gargalovic P.S., Lusis A.J. (2009). CHAC1/MGC4504 is a novel proapoptotic component of the unfolded protein response, downstream of the ATF4-ATF3-CHOP cascade. J. Immunol..

[25] Lash L.H. (2006). Mitochondrial glutathione transport: Physiological, pathological and toxicological implications. Chem. Biol. Interact..

[26] Bánhegyi G., Csala M., Nagy G., Sorrentino V., Fulceri R., Benedetti A. (2003). Evidence for the transport of glutathione through ryanodine receptor channel type 1. Biochem. J..

[27] Csala M., Fulceri R., Mandl J., Benedetti A., Bánhegyi G. (2001). Ryanodine receptor channel-dependent glutathione transport in the sarcoplasmic reticulum of skeletal muscle. Biochem. Biophys. Res. Commun..

[28] Belcastro E., Wu W., Fries-Raeth I., Corti A., Pompella A., Leroy P., Lartaud I., Gaucher C. (2017). Oxidative stress enhances and modulates protein S-nitrosation in smooth muscle cells exposed to S-nitrosoglutathione. Nitric Oxide Biol. Chem..

[29] Ballatori N., Krance S.M., Marchan R., Hammond C.L. (2009). Plasma membrane glutathione transporters and their roles in cell physiology and pathophysiology. Mol. Asp. Med..

[30] Muanprasat C., Wongborisuth C., Pathomthongtaweechai N., Satitsri S., Hongeng S. (2013). Protection against oxidative stress in beta thalassemia/hemoglobin E erythrocytes by inhibitors of glutathione efflux transporters. PLoS ONE.

[31] Dickinson D.A., Forman H.J. (2002). Cellular glutathione and thiols metabolism. Biochem. Pharmacol..

[32] Forman H.J., Zhang H., Rinna A. (2009). Glutathione: Overview of its protective roles, measurement, and biosynthesis. Mol. Asp. Med..

[33] Schafer F.Q., Buettner G.R. (2001). Redox environment of the cell as viewed through the redox state of the glutathione disulfide/glutathione couple. Free Radic. Biol. Med..

[34] Nagy P. (2013). Kinetics and mechanisms of thiol-disulfide exchange covering direct substitution and thiol-oxidation pathways. Antioxid. Redox Signal..

[35] Paolicchi A., Dominici S., Pieri L., Maellaro E., Pompella A. (2002). Glutathione catabolism as a signaling mechanism. Biochem. Pharmacol..

[36] Dominici S., Paolicchi A., Corti A., Maellaro E., Pompella A. (2005). Prooxidant reactions promoted by soluble and cell-bound gamma-glutamyltransferase activity. Methods Enzymol..

[37] Drozdz R., Parmentier C., Hachad H., Leroy P., Siest G., Wellman M. (1998). gamma-Glutamyltransferase dependent generation of reactive oxygen species from a glutathione/transferrin system. Free Radic. Biol. Med..

[38] Dominici S., Pieri L., Comporti M., Pompella A. (2003). Possible role of membrane gamma-glutamyltransferase activity in the facilitation of transferrin-dependent and -independent iron uptake by cancer cells. Cancer Cell Int..

[39] Glass G.A., Stark A.A. (1997). Promotion of glutathione-gamma-glutamyl transpeptidase-dependent lipid peroxidation by copper and ceruloplasmin: The requirement for iron and the effects of antioxidants and antioxidant enzymes. Environ. Mol. Mutagen..

[40] Schmidt A.M., Hori O., Brett J., Yan S.D., Wautier J.L., Stern D. (1994). Cellular receptors for advanced glycation end products. Implications for induction of oxidant stress and cellular dysfunction in the pathogenesis of vascular lesions. Arterioscler. Thromb. J. Vasc. Biol..

[41] Gimbrone M.A. (1995). Vascular endothelium: An integrator of pathophysiologic stimuli in atherosclerosis. Am. J. Cardiol..

[42] Ross R. (1993). The pathogenesis of atherosclerosis: A perspective for the 1990s. Nature.

[43] Bradley J.R. (2008). TNF-mediated inflammatory disease. J. Pathol..

[44] Paolicchi A., Minotti G., Tonarelli P., Tongiani R., De Cesare D., Mezzetti A., Dominici S., Comporti M., Pompella A. (1999). Gamma-glutamyl transpeptidase-dependent iron reduction and LDL oxidation—A potential mechanism in atherosclerosis. J. Investig. Med..

[45] Berliner J.A., Heinecke J.W. (1996). The role of oxidized lipoproteins in atherogenesis. Free Radic. Biol. Med..

[46] Jones D.P. (2002). Redox potential of GSH/GSSG couple: Assay and biological significance. Methods Enzymol..

[47] Sen C.K. (2000). Cellular thiols and redox-regulated signal transduction. Curr. Top. Cell. Regul..

[48] Conour J.E., Graham W.V., Gaskins H.R. (2004). A combined in vitro/bioinformatic investigation of redox regulatory mechanisms governing cell cycle progression. Physiol. Genom..

[49] Menon S.G., Sarsour E.H., Spitz D.R., Higashikubo R., Sturm M., Zhang H., Goswami P.C. (2003). Redox regulation of the G1 to S phase transition in the mouse embryo fibroblast cell cycle. Cancer Res..

[50] Menon S.G., Sarsour E.H., Kalen A.L., Venkataraman S., Hitchler M.J., Domann F.E., Oberley L.W., Goswami P.C. (2007). Superoxide signaling mediates N-acetyl-L-cysteine-induced G1 arrest: Regulatory role of cyclin D1 and manganese superoxide dismutase. Cancer Res..

[51] Belcastro E., Gaucher C., Corti A., Leroy P., Lartaud I., Pompella A. (2017). Regulation of protein function by S-nitrosation and S-glutathionylation: Processes and targets in cardiovascular pathophysiology. Biol. Chem..

[52] Holmgren A. (2000). Redox regulation by thioredoxin and thioredoxin reductase. BioFactors.

[53] Pastore A., Federici G., Bertini E., Piemonte F. (2003). Analysis of glutathione: Implication in redox and detoxification. Clin. Chim. Acta.

[54] Schulz J.B., Lindenau J., Seyfried J., Dichgans J. (2000). Glutathione, oxidative stress and neurodegeneration. Eur. J. Biochem..

[55] Anderson M.F., Nilsson M., Eriksson P.S., Sims N.R. (2004). Glutathione monoethyl ester provides neuroprotection in a rat model of stroke. Neurosci. Lett..

[56] Vargas F., Rodríguez-Gómez I., Pérez-Abud R., Vargas Tendero P., Baca Y., Wangensteen R. (2012). Cardiovascular and renal manifestations of glutathione depletion induced by buthionine sulfoximine. Am. J. Hypertens..

[57] Beutler E., Duron O., Kelly B.M. (1963). Improved method for the determination of blood glutathione. J. Lab. Clin. Med..

[58] Mansoor M.A., Svardal A.M., Ueland P.M. (1992). Determination of the in vivo redox status of cysteine, cysteinylglycine, homocysteine, and glutathione in human plasma. Anal. Biochem..

[59] Tietze F. (1969). Enzymic method for quantitative determination of nanogram amounts of total and oxidized glutathione: Applications to mammalian blood and other tissues. Anal. Biochem..

[60] Chen Z., Wang Z., Chen J., Wang S., Huang X. (2012). Sensitive and selective detection of glutathione based on resonance light scattering using sensitive gold nanoparticles as colorimetric probes. Analyst.

[61] Xu H., Hepel M. (2011). “Molecular beacon”—Based fluorescent assay for selective detection of glutathione and cysteine. Anal. Chem..

[62] Piccoli G., Fiorani M., Biagiarelli B., Palma F., Potenza L., Amicucci A., Stocchi V. (1994). Simultaneous high-performance capillary electrophoretic determination of reduced and oxidized glutathione in red blood cells in the femtomole range. J. Chromatogr. A.

[63] Park S.K., Boulton R.B., Noble A.C. (2000). Automated HPLC analysis of glutathione and thiol-containing compounds in grape juice and wine using pre-column derivatization with fluorescence detection. Food Chem..

[64] Parmentier C., Leroy P., Wellman M., Nicolas A. (1998). Determination of cellular thiols and glutathione-related enzyme activities: Versatility of high-performance liquid chromatography-spectrofluorimetric detection. J. Chromatogr. B. Biomed. Sci. Appl..

[65] Parmentier C., Wellman M., Nicolas A., Siest G., Leroy P. (1999). Simultaneous measurement of reactive oxygen species and reduced glutathione using capillary electrophoresis and laser-induced fluorescence detection in cultured cell lines. Electrophoresis.

[66] Lewicki K., Marchand S., Matoub L., Lulek J., Coulon J., Leroy P. (2006). Development of a fluorescence-based microtiter plate method for the measurement of glutathione in yeast. Talanta.

[67] Rezaei B., Khosropour H., Ensafi A.A., Hadadzadeh H., Farrokhpour H. (2015). A Differential Pulse Voltammetric Sensor for Determination of Glutathione in Real Samples Using a Trichloro(terpyridine)ruthenium(III)/Multiwall Carbon Nanotubes Modified Paste Electrode. IEEE Sens. J..

[68] Burford N., Eelman M.D., Mahony D.E., Morash M. (2003). Definitive identification of cysteine and glutathione complexes of bismuth by mass spectrometry: Assessing the biochemical fate of bismuth pharmaceutical agents. Chem. Commun..

[69] Han H.-Y., He Z.-K., Zeng Y.-E. (2006). Chemiluminescence method for the determination of glutathione in human serum using the Ru(phen)_3_^2+^-KMnO_4_ system. Microchim. Acta.

[70] Mandal P.K., Tripathi M., Sugunan S. (2012). Brain oxidative stress: Detection and mapping of anti-oxidant marker “Glutathione” in different brain regions of healthy male/female, MCI and Alzheimer patients using non-invasive magnetic resonance spectroscopy. Biochem. Biophys. Res. Commun..

[71] Huang G.G., Hossain M.K., Han X.X., Ozaki Y. (2009). A novel reversed reporting agent method for surface-enhanced Raman scattering; highly sensitive detection of glutathione in aqueous solutions. Analyst.

[72] Vallverdú-Queralt A., Verbaere A., Meudec E., Cheynier V., Sommerer N. (2015). Straightforward method to quantify GSH, GSSG, GRP, and hydroxycinnamic acids in wines by UPLC-MRM-MS. J. Agric. Food Chem..

[73] Parent M., Dahboul F., Schneider R., Clarot I., Maincent P., Leroy P., Boudier A. A Complete Physicochemical Identity Card of S-Nitrosoglutathione. http://www.eurekaselect.com/105996/article.

[74] Neuschwander-Tetri B.A., Roll F.J. (1989). Glutathione measurement by high-performance liquid chromatography separation and fluorometric detection of the glutathione-orthophthalaldehyde adduct. Anal. Biochem..

[75] Serru V., Baudin B., Ziegler F., David J.P., Cals M.J., Vaubourdolle M., Mario N. (2001). Quantification of reduced and oxidized glutathione in whole blood samples by capillary electrophoresis. Clin. Chem..

[76] European Department for the Quality of Medicines (2018). Glutathione, Monograph 01/2017: 1670, European Pharmacopoeia.

[77] Pensa E., Cortés E., Corthey G., Carro P., Vericat C., Fonticelli M.H., Benítez G., Rubert A.A., Salvarezza R.C. (2012). The chemistry of the sulfur-gold interface: In search of a unified model. Acc. Chem. Res..

[78] Lin Z., Monteiro-Riviere N.A., Riviere J.E. (2015). Pharmacokinetics of metallic nanoparticles. Wiley Interdiscip. Rev. Nanomed. Nanobiotechnol..

[79] Bhattacharjee A., Chakraborty K., Shukla A. (2017). Cellular copper homeostasis: Current concepts on its interplay with glutathione homeostasis and its implication in physiology and human diseases. Metallomics.

[80] Reimers J.R., Ford M.J., Marcuccio S.M., Ulstrup J., Hush N.S. (2017). Competition of van der Waals and chemical forces on gold–sulfur surfaces and nanoparticles. Nat. Rev. Chem..

[81] Tsogas G.Z., Kappi F.A., Vlessidis A.G., Giokas D.L. (2018). Recent Advances in Nanomaterial Probes for Optical Biothiol Sensing: A Review. Anal. Lett..

[82] Li Z.-J., Zheng X.-J., Zhang L., Liang R.-P., Li Z.-M., Qiu J.-D. (2015). Label-free colorimetric detection of biothiols utilizing SAM and unmodified Au nanoparticles. Biosens. Bioelectron..

[83] Li J.-F., Huang P.-C., Wu F.-Y. (2017). Highly selective and sensitive detection of glutathione based on anti-aggregation of gold nanoparticles via pH regulation. Sens. Actuators B Chem..

[84] Shen C.-C., Tseng W.-L., Hsieh M.-M. (2009). Selective enrichment of aminothiols using polysorbate 20-capped gold nanoparticles followed by capillary electrophoresis with laser-induced fluorescence. J. Chromatogr. A.

[85] Shen L.-M., Chen Q., Sun Z.-Y., Chen X.-W., Wang J.-H. (2014). Assay of biothiols by regulating the growth of silver nanoparticles with C-dots as reducing agent. Anal. Chem..

[86] Kappi F.A., Papadopoulos G.A., Tsogas G.Z., Giokas D.L. (2017). Low-cost colorimetric assay of biothiols based on the photochemical reduction of silver halides and consumer electronic imaging devices. Talanta.

[87] Dringen R., Koehler Y., Derr L., Tomba G., Schmidt M.M., Treccani L., Colombi Ciacchi L., Rezwan K. (2011). Adsorption and reduction of glutathione disulfide on α-Al_2_O_3_ nanoparticles: Experiments and modeling. Langmuir.

[88] Barman U., Mukhopadhyay G., Goswami N., Ghosh S.S., Paily R.P. (2017). Detection of Glutathione by Glutathione-S-Transferase-Nanoconjugate Ensemble Electrochemical Device. IEEE Trans. Nanobiosci..

[89] Estrela J., Obrador E., Navarro J., Delavega M., Pellicer J. (1995). Elimination of Ehrlich Tumors by ATP-Induced Growth-Inhibition, Glutathione Depletion and X-Rays. Nat. Med..

[90] Mena S., Benlloch M., Ortega A., Carretero J., Obrador E., Asensi M., Petschen I., Brown B.D., Estrela J.M. (2007). Bcl-2 and glutathione depletion sensitizes B16 melanoma to combination therapy and eliminates metastatic disease. Clin. Cancer Res..

[91] Rocha C.R.R., Garcia C.C.M., Vieira D.B., Quinet A., de Andrade-Lima L.C., Munford V., Belizario J.E., Menck C.F.M. (2014). Glutathione depletion sensitizes cisplatin- and temozolomide-resistant glioma cells in vitro and in vivo. Cell Death Dis..

[92] Lagman M., Ly J., Saing T., Kaur Singh M., Vera Tudela E., Morris D., Chi P.-T., Ochoa C., Sathananthan A., Venketaraman V. (2015). Investigating the causes for decreased levels of glutathione in individuals with type II diabetes. PLoS ONE.

[93] Herzenberg L.A., DeRosa S.C., Dubs J.G., Roederer M., Anderson M.T., Ela S.W., Deresinski S.C., Herzenberg L.A. (1997). Glutathione deficiency is associated with impaired survival in HIV disease. Proc. Natl. Acad. Sci. USA.

[94] Biolo G., Antonione R., De Cicco M. (2007). Glutathione metabolism in sepsis. Crit. Care Med..

[95] Day B.J. (2005). Glutathione—A radical treatment for cystic fibrosis lung disease?. Chest.

[96] Martin H.L., Teismann P. (2009). Glutathione—A review on its role and significance in Parkinson’s disease. FASEB J..

[97] Pocernich C.B., Butterfield D.A. (2012). Elevation of glutathione as a therapeutic strategy in Alzheimer disease. Biochim. Biophys. Acta.

[98] Gu F., Chauhan V., Chauhan A. (2015). Glutathione redox imbalance in brain disorders. Curr. Opin. Clin. Nutr. Metab. Care.

[99] Jones D.P., Mody V.C., Carlson J.L., Lynn M.J., Sternberg P. (2002). Redox analysis of human plasma allows separation of pro-oxidant events of aging from decline in antioxidant defenses. Free Radic. Biol. Med..

[100] Witschi A., Reddy S., Stofer B., Lauterburg B.H. (1992). The systemic availability of oral glutathione. Eur. J. Clin. Pharmacol..

[101] Shibui Y., Sakai R., Manabe Y., Masuyama T. (2017). Comparisons of l-cysteine and d-cysteine toxicity in 4-week repeated-dose toxicity studies of rats receiving daily oral administration. J. Toxicol. Pathol..

[102] Greene S.C., Noonan P.K., Sanabria C., Peacock W.F. (2016). Effervescent N-Acetylcysteine Tablets versus Oral Solution N-Acetylcysteine in Fasting Healthy Adults: An Open-Label, Randomized, Single-Dose, Crossover, Relative Bioavailability Study. Curr. Ther. Res. Clin. Exp..

[103] Dröge W., Breitkreutz R. (1999). N-acetyl-cysteine in the therapy of HIV-positive patients. Curr. Opin. Clin. Nutr. Metab. Care.

[104] Borgström L., Kågedal B., Paulsen O. (1986). Pharmacokinetics of N-acetylcysteine in man. Eur. J. Clin. Pharmacol..

[105] Grinberg L., Fibach E., Amer J., Atlas D. (2005). N-acetylcysteine amide, a novel cell-permeating thiol, restores cellular glutathione and protects human red blood cells from oxidative stress. Free Radic. Biol. Med..

[106] Patel S.P., Sullivan P.G., Pandya J.D., Goldstein G.A., VanRooyen J.L., Yonutas H.M., Eldahan K.C., Morehouse J., Magnuson D.S.K., Rabchevsky A.G. (2014). N-acetylcysteine amide preserves mitochondrial bioenergetics and improves functional recovery following spinal trauma. Exp. Neurol..

[107] Giustarini D., Milzani A., Dalle-Donne I., Tsikas D., Rossi R. (2012). N-Acetylcysteine ethyl ester (NACET): A novel lipophilic cell-permeable cysteine derivative with an unusual pharmacokinetic feature and remarkable antioxidant potential. Biochem. Pharmacol..

[108] Anderson M.E., Powrie F., Puri R.N., Meister A. (1985). Glutathione monoethyl ester: Preparation, uptake by tissues, and conversion to glutathione. Arch. Biochem. Biophys..

[109] Grattagliano I., Wieland P., Schranz C., Lauterburg B.H. (1994). Effect of oral glutathione monoethyl ester and glutathione on circulating and hepatic sulfhydrils in the rat. Pharmacol. Toxicol..

[110] Minhas H.S., Thornalley P.J. (1995). Comparison of the delivery of reduced glutathione into P388D1 cells by reduced glutathione and its mono- and diethyl ester derivatives. Biochem. Pharmacol..

[111] Zampagni M., Wright D., Cascella R., D’Adamio G., Casamenti F., Evangelisti E., Cardona F., Goti A., Nacmias B., Sorbi S. (2012). Novel S-acyl glutathione derivatives prevent amyloid oxidative stress and cholinergic dysfunction in Alzheimer disease models. Free Radic. Biol. Med..

[112] Lieber C.S. (2002). S-Adenosyl-L-methionine and alcoholic liver disease in animal models: Implications for early intervention in human beings. Alcohol.

[113] Fawcett J.P., Schiller B., Jiang R., Moran J., Walker R.J. (1996). Supplementation with L-2-oxothiazolidine-4-carboxylic acid, a cysteine precursor, does not protect against lipid peroxidation in puromycin aminonucleoside-induced nephropathy. Exp. Nephrol..

[114] Oz H.S., Chen T.S., Nagasawa H. (2007). Comparative efficacies of 2 cysteine prodrugs and a glutathione delivery agent in a colitis model. Transl. Res. J. Lab. Clin. Med..

[115] Zarka M.H., Bridge W.J. (2017). Oral administration of γ-glutamylcysteine increases intracellular glutathione levels above homeostasis in a randomised human trial pilot study. Redox Biol..

[116] Rosenblat M., Volkova N., Coleman R., Aviram M. (2007). Anti-oxidant and anti-atherogenic properties of liposomal glutathione: Studies in vitro, and in the atherosclerotic apolipoprotein E-deficient mice. Atherosclerosis.

[117] Wen J., Du Y., Li D., Alany R. (2013). Development of water-in-oil microemulsions with the potential of prolonged release for oral delivery of L-glutathione. Pharm. Dev. Technol..

[118] Baek M., Choy J.-H., Choi S.-J. (2012). Montmorillonite intercalated with glutathione for antioxidant delivery: Synthesis, characterization, and bioavailability evaluation. Int. J. Pharm..

[119] Trapani A., Laquintana V., Denora N., Lopedota A., Cutrignelli A., Franco M., Trapani G., Liso G. (2007). Eudragit RS 100 microparticles containing 2-hydroxypropyl-β-cyclodextrin and glutathione: Physicochemical characterization, drug release and transport studies. Eur. J. Pharm. Sci..

[120] Trapani A., Lopedota A., Franco M., Cioffi N., Ieva E., Garcia-Fuentes M., Alonso M.J. (2010). A comparative study of chitosan and chitosan/cyclodextrin nanoparticles as potential carriers for the oral delivery of small peptides. Eur. J. Pharm. Biopharm..

[121] Naji-Tabasi S., Razavi S.M.A., Mehditabar H. (2017). Fabrication of basil seed gum nanoparticles as a novel oral delivery system of glutathione. Carbohydr. Polym..

[122] Mandracchia D., Denora N., Franco M., Pitarresi G., Giammona G., Trapani G. (2011). New Biodegradable Hydrogels Based on Inulin and alpha,beta-Polyaspartylhydrazide Designed for Colonic Drug Delivery: In Vitro Release of Glutathione and Oxytocin. J. Biomater. Sci. Polym. Ed..

[123] Chen G., Bunt C., Wen J. (2015). Mucoadhesive polymers-based film as a carrier system for sublingual delivery of glutathione. J. Pharm. Pharmacol..

[124] Buonocore D., Grosini M., Giardina S., Michelotti A., Carrabetta M., Seneci A., Verri M., Dossena M., Marzatico F. (2016). Bioavailability Study of an Innovative Orobuccal Formulation of Glutathione. Oxid. Med. Cell. Longev..

[125] Beqa L., Singh A.K., Khan S.A., Senapati D., Arumugam S.R., Ray P.C. (2011). Gold nanoparticle-based simple colorimetric and ultrasensitive dynamic light scattering assay for the selective detection of Pb(II) from paints, plastics, and water samples. ACS Appl. Mater. Interfaces.

[126] Zhang Z., Jia J., Lai Y., Ma Y., Weng J., Sun L. (2010). Conjugating folic acid to gold nanoparticles through glutathione for targeting and detecting cancer cells. Bioorg. Med. Chem..

[127] Valusová E., Svec P., Antalík M. (2009). Structural and thermodynamic behavior of cytochrome c assembled with glutathione-covered gold nanoparticles. J. Biol. Inorg. Chem..

[128] Polavarapu L., Manna M., Xu Q.-H. (2011). Biocompatible glutathione capped gold clusters as one- and two-photon excitation fluorescence contrast agents for live cells imaging. Nanoscale.

[129] Simpson C.A., Salleng K.J., Cliffel D.E., Feldheim D.L. (2013). In vivo toxicity, biodistribution, and clearance of glutathione-coated gold nanoparticles. Nanomed. Nanotechnol. Biol. Med..

[130] Koo S.H., Lee J.-S., Kim G.-H., Lee H.G. (2011). Preparation, Characteristics, and Stability of Glutathione-Loaded Nanoparticles. J. Agric. Food Chem..

[131] Williams S.R., Lepene B.S., Thatcher C.D., Long T.E. (2009). Synthesis and characterization of poly(ethylene glycol)-glutathione conjugate self-assembled nanoparticles for antioxidant delivery. Biomacromolecules.

[132] Tortiglione C., Quarta A., Tino A., Manna L., Cingolani R., Pellegrino T. (2007). Synthesis and biological assay of GSH functionalized fluorescent quantum dots for staining Hydra vulgaris. Bioconjug. Chem..

[133] Tournebize J., Boudier A., Sapin-Minet A., Maincent P., Leroy P., Schneider R. (2012). Role of gold nanoparticles capping density on stability and surface reactivity to design drug delivery platforms. ACS Appl. Mater. Interfaces.

[134] Tournebize J., Boudier A., Joubert O., Eidi H., Bartosz G., Maincent P., Leroy P., Sapin-Minet A. (2012). Impact of gold nanoparticle coating on redox homeostasis. Int. J. Pharm..

[135] Luo M., Boudier A., Clarot I., Maincent P., Schneider R., Leroy P. (2016). Gold Nanoparticles Grafted by Reduced Glutathione With Thiol Function Preservation. Colloid Interface Sci. Commun..

[136] Levin V.A. (1980). Relationship of octanol/water partition coefficient and molecular weight to rat brain capillary permeability. J. Med. Chem..

[137] Kannan R., Kuhlenkamp J.F., Jeandidier E., Trinh H., Ookhtens M., Kaplowitz N. (1990). Evidence for carrier-mediated transport of glutathione across the blood-brain barrier in the rat. J. Clin. Investig..

[138] Zlokovic B.V., Mackic J.B., McComb J.G., Weiss M.H., Kaplowitz N., Kannan R. (1994). Evidence for transcapillary transport of reduced glutathione in vascular perfused guinea-pig brain. Biochem. Biophys. Res. Commun..

[139] Kannan R., Chakrabarti R., Tang D., Kim K.J., Kaplowitz N. (2000). GSH transport in human cerebrovascular endothelial cells and human astrocytes: Evidence for luminal localization of Na+-dependent GSH transport in HCEC. Brain Res..

[140] Rip J., Chen L., Hartman R., van den Heuvel A., Reijerkerk A., van Kregten J., van der Boom B., Appeldoorn C., de Boer M., Maussang D. (2014). Glutathione PEGylated liposomes: Pharmacokinetics and delivery of cargo across the blood-brain barrier in rats. J. Drug Target..

[141] Rotman M., Welling M.M., Bunschoten A., de Backer M.E., Rip J., Nabuurs R.J.A., Gaillard P.J., van Buchem M.A., van der Maarel S.M., van der Weerd L. (2015). Enhanced glutathione PEGylated liposomal brain delivery of an anti-amyloid single domain antibody fragment in a mouse model for Alzheimer’s disease. J. Control. Release.

[142] Maussang D., Rip J., van Kregten J., van den Heuvel A., van der Pol S., van der Boom B., Reijerkerk A., Chen L., de Boer M., Gaillard P. (2016). Glutathione conjugation dose-dependently increases brain-specific liposomal drug delivery in vitro and in vivo. Drug Discov. Today Technol..

[143] Birngruber T., Raml R., Gladdines W., Gatschelhofer C., Gander E., Ghosh A., Kroath T., Gaillard P.J., Pieber T.R., Sinner F. (2014). Enhanced doxorubicin delivery to the brain administered through glutathione PEGylated liposomal doxorubicin (2B3-101) as compared with generic Caelyx,(®)/Doxil(®)—A cerebral open flow microperfusion pilot study. J. Pharm. Sci..

[144] Gaillard P.J., Appeldoorn C.C.M., Dorland R., van Kregten J., Manca F., Vugts D.J., Windhorst B., van Dongen G.A.M.S., de Vries H.E., Maussang D. (2014). Pharmacokinetics, brain delivery, and efficacy in brain tumor-bearing mice of glutathione pegylated liposomal doxorubicin (2B3-101). PLoS ONE.

[145] Gaillard P.J., Appeldoorn C.C.M., Rip J., Dorland R., van der Pol S.M.A., Kooij G., de Vries H.E., Reijerkerk A. (2012). Enhanced brain delivery of liposomal methylprednisolone improved therapeutic efficacy in a model of neuroinflammation. J. Control. Release.

[146] Kanhai K.M.S., Zuiker R.G.J.A., Stavrakaki I., Gladdines W., Gaillard P.J., Klaassen E.S., Groeneveld G.J. Glutathione-PEGylated liposomal methylprednisolone in comparison to free methylprednisolone: Slow release characteristics and prolonged lymphocyte depression in a first-in-human study. Br. J. Clin. Pharmacol..

[147] Gaillard P.J., Kerklaan B.M., Aftimos P., Altintas S., Jager A., Gladdines W., Lonnqvist F., Soetekouw P., Verheul H., Awada A. (2014). Abstract CT216: Phase I dose escalating study of 2B3-101, glutathione PEGylated liposomal doxorubicin, in patients with solid tumors and brain metastases or recurrent malignant glioma. Cancer Res..

[148] Brandsma D., Kerklaan B.M., Diéras V., Altintas S., Anders C.K., Ballester M.A., Gelderblom H., Soetekouw P.M.M.B., Gladdines W., Lonnqvist F. (2014). Phase 1/2a study of glutathione pegylated liposomal doxorubicin (2b3-101) in patients with brain metastases (BM) from solid tumors or recurrent high grade gliomas (HGG). Ann. Oncol..

[149] Veszelka S., Meszaros M., Kiss L., Kota Z., Pali T., Hoyk Z., Bozso Z., Fulop L., Toth A., Rakhely G. (2017). Biotin and Glutathione Targeting of Solid Nanoparticles to Cross Human Brain Endothelial Cells. Curr. Pharm. Des..

[150] Grover A., Hirani A., Pathak Y., Sutariya V. (2014). Brain-targeted delivery of docetaxel by glutathione-coated nanoparticles for brain cancer. AAPS PharmSciTech.

[151] Geldenhuys W., Wehrung D., Groshev A., Hirani A., Sutariya V. (2015). Brain-targeted delivery of doxorubicin using glutathione-coated nanoparticles for brain cancers. Pharm. Dev. Technol..

[152] Geldenhuys W., Mbimba T., Bui T., Harrison K., Sutariya V. (2011). Brain-targeted delivery of paclitaxel using glutathione-coated nanoparticles for brain cancers. J. Drug Target..

[153] Raval N., Mistry T., Acharya N., Acharya S. (2015). Development of glutathione-conjugated asiatic acid-loaded bovine serum albumin nanoparticles for brain-targeted drug delivery. J. Pharm. Pharmacol..

[154] Patel P., Acharya N., Acharya S. (2013). Development and characterization of glutathione-conjugated albumin nanoparticles for improved brain delivery of hydrophilic fluorescent marker. Drug Deliv..

[155] Mdzinarishvili A., Sutariya V., Talasila P.K., Geldenhuys W.J., Sadana P. (2013). Engineering triiodothyronine (T3) nanoparticle for use in ischemic brain stroke. Drug Deliv. Transl. Res..

[156] Englert C., Trützschler A.-K., Raasch M., Bus T., Borchers P., Mosig A.S., Traeger A., Schubert U.S. (2016). Crossing the blood-brain barrier: Glutathione-conjugated poly(ethylene imine) for gene delivery. J. Control. Release.

[157] Yin J., Chen Y., Zhang Z.-H., Han X. (2016). Stimuli-Responsive Block Copolymer-Based Assemblies for Cargo Delivery and Theranostic Applications. Polymers.

[158] Liu X., Yang Y., Urban M.W. (2017). Stimuli-Responsive Polymeric Nanoparticles. Macromol. Rapid Commun..

[159] Cheng R., Meng F., Deng C., Klok H.-A., Zhong Z. (2013). Dual and multi-stimuli responsive polymeric nanoparticles for programmed site-specific drug delivery. Biomaterials.

[160] Son S., Namgung R., Kim J., Singha K., Kim W.J. (2012). Bioreducible polymers for gene silencing and delivery. Acc. Chem. Res..

[161] Ma N., Li Y., Xu H., Wang Z., Zhang X. (2010). Dual redox responsive assemblies formed from diselenide block copolymers. J. Am. Chem. Soc..

[162] Han L., Zhang X.-Y., Wang Y.-L., Li X., Yang X.-H., Huang M., Hu K., Li L.-H., Wei Y. (2017). Redox-responsive theranostic nanoplatforms based on inorganic nanomaterials. J. Control. Release.

[163] Ghosh P.S., Kim C.-K., Han G., Forbes N.S., Rotello V.M. (2008). Efficient Gene Delivery Vectors by Tuning the Surface Charge Density of Amino Acid-Functionalized Gold Nanoparticles. ACS Nano.

[164] Tournebize J., Sapin-Minet A., Bartosz G., Leroy P., Boudier A. (2013). Pitfalls of assays devoted to evaluation of oxidative stress induced by inorganic nanoparticles. Talanta.

